# Caloric Restriction and the Nutrient-Sensing PGC-1**α** in Mitochondrial Homeostasis: New Perspectives in Neurodegeneration

**DOI:** 10.1155/2012/759583

**Published:** 2012-07-08

**Authors:** Daniele Lettieri Barbato, Sara Baldelli, Beatrice Pagliei, Katia Aquilano, Maria Rosa Ciriolo

**Affiliations:** ^1^Department of Biology, University of Rome “Tor Vergata”, Via della Ricerca Scientifica 1, 00133 Rome, Italy; ^2^Research Center, IRCCS San Raffaele La Pisana, Via di Val Cannuta 247, 00166 Rome, Italy

## Abstract

Mitochondrial activity progressively declines during ageing and in many neurodegenerative diseases. Caloric restriction (CR) has been suggested as a dietary intervention that is able to postpone the detrimental aspects of aging as it ameliorates mitochondrial performance. This effect is partially due to increased mitochondrial biogenesis. The nutrient-sensing PGC-1*α* is a transcriptional coactivator that promotes the expression of mitochondrial genes and is induced by CR. It is believed that many of the mitochondrial and metabolic benefits of CR are due to increased PGC-1*α* activity. The increase of PGC-1*α* is also positively linked to neuroprotection and its decrement has been involved in the pathogenesis of many neurodegenerative diseases. This paper aims to summarize the current knowledge about the role of PGC-1*α* in neuronal homeostasis and the beneficial effects of CR on mitochondrial biogenesis and function. We also discuss how PGC-1*α*-governed pathways could be used as target for nutritional intervention to prevent neurodegeneration.

## 1. Introduction

Mitochondria are dynamic organelles fundamental for cell life. The central roles of mitochondria in metabolism place them at the center stage of global energy modulation. Indeed, these organelles are best known for producing ATP via oxidative phosphorylation (OXPHOS). In the matrix, tricarboxylic acid cycle (TCA) generates reduced carriers (NADH and FADH2), which donate electrons to the inner membrane-localized electron transport chain (ETC). In this way electrons derived from metabolites flow through the ETC generating intermembrane proton gradient (mitochondrial membrane potential), which fundamental for the activity of rotary turbine-like ATP synthase producing ATP from ADP [1].

Mitochondria contain a circular genome, mitochondrial DNA (mtDNA), which has been reduced during evolution through gene transfer to the nucleus. Even if mitochondria are characterized by an own genome encoding for 13 subunits of ETC, they strongly necessitate of many other nuclear encoded proteins (about 1000–1500) for the execution of their function [2]. Therefore, it is clear that mitochondria are not autonomous entities but strongly dependent on nuclear genome. Indeed, mitochondrial homeostasis is assured by a well-functioning bidirectional network of mitochondrial-nuclear communications (MN-C). MN-C control many cell activities including mitochondrial biogenesis, an intricate biological process consisting in the growth and division of preexisting mitochondria that requires the replication of the mtDNA and the synthesis and import of proteins and lipids to the existing mitochondria [3]. Mitochondrial biogenesis is substantially driven by the nuclear genome, where an interconnected network of transcription factors regulates the expression of mitochondrial proteins including those that control replication and transcription of mtDNA. All these processes have to be finely regulated and coordinated in response to a wide range of physiological cues that affect cellular energetic need (e.g., temperature, physical activity, and nutrient availability). NAD^+^ and AMP are considered key second messengers for nuclear-mitochondrial communications and hence for mitochondrial metabolic adaptation and mitochondrial biogenesis. They orchestrate an integrated physiological response through the specific activation of molecular effectors including transcription factors, cofactors, nuclear receptors, and kinases. Important cellular sensors of metabolic status enrolled into mitochondrial-nuclear axis are: (i) the AMP-activated protein kinase (AMPK), (ii) the NAD^+^-dependent deacetylase sirtuin 1 (SIRT1), and (iii) the peroxisome proliferator-activated receptor gamma coactivator-1 alpha (PGC-1*α*). AMPK is activated by an increase in AMP : ATP ratio and increased ADP concentrations, both of which are linked to an energetic drop [4–6]. SIRT1 responds to elevated levels of NAD^+^ that occur upon starvation and acts in synergy with AMPK in regulating mitochondrial mass, nutrient oxidation, and ATP production to fit cells needs via the transcriptional coactivator PGC-1*α* [6, 7]. Mitochondrial also represent the principal source of reactive oxygen species (ROS), which are considered important second messengers orchestrating MN-C. Many reports have showed that ROS can enhance the transcription of genes responsible for the growth of many cellular types [8]. In particular, in cancer cells the high proliferation rate is associated with an increased oxidative intracellular environment due to dysbalance of mitochondrial ROS production [9]. A key role in the MN-C is also played by nitric oxide (NO), a small signaling molecule that in brain functions as a neurotransmitter and neuromodulator, exerting a regulatory effect on neuronal function. Even though the production of NO by mitochondria is still a debated matter of research [10], it is now clear that this second messenger is able to dictate the shuttling and/or activation of redox-sensitive transcription factors to the nucleus. In doing so, similarly to ROS, NO redirects gene expression for adaptation of cells to stress or commitment to death. Physiological concentration of NO may efficiently buffer cell death induced by antiproliferative agents dampening the activity of the mitochondrial apoptotic cascade governed by the JNK/c-Jun signaling pathway [11]. On the contrary high concentration of NO can inhibit cell proliferation via the activation of p53 and/or induce caspase-independent apoptosis mediated by the shuttling of AIF from the mitochondria to the nucleus [12, 13]. The uncontrolled increase of ROS/NO flux is considered a contributing pathogenic factor in the ageing process and in many human diseases [14, 15]. The oxidative/nitrosative stress caused by the increase of mitochondrial ROS as well as upregulation of NO synthases are commonly observed in many neurodegenerative diseases including Parkinson's disease (PD) [16]. It is worth noting that also physiological flux of NO can become detrimental for neuronal cells in condition of limited antioxidant availability. In particular, we have showed that the decline of glutathione (GSH), the most abundant nonenzymatic thiol antioxidant, is the primary cause of endogenous NO neurotoxicity, that is, characterized by a prominent protein damage via S-nitrosylation and nitration [13, 17]. In line with this evidence, also the decrease of enzymatic antioxidant for example, SOD1, result in a ROS-mediated damaging effect on cellular targets including mitochondria specifically in cells of neuronal origin [18, 19]. Therefore, the decline of antioxidant levels, generally occurring during physiological ageing, may be likely considered a key factor triggering NO/ROS-mediated neuronal death in ageing and neurodegenerative-related diseases.

Among cellular systems, neurons are exquisitely dependent upon mitochondria to support their high energy-demanding functions [20]. The intricate role of mitochondria in maintaining neuronal metabolism emerges from the causal relationship between their impairment and several neurological disorders. In neurons, mitochondria supply substantial amounts of ATP as well as TCA intermediates that serve as the building blocks for synthesis of neurotransmitters [21, 22]. For instance it has been shown that pyruvate carboxylase deficiency strongly alters brain amino acid content affecting the release of gamma-aminobutyric acid (GABA) and glutamate neurotransmitters [23]. Mitochondria-mediated lipid synthesis is also critical for neuronal function, as defects in lipoic acid synthase cause severe neonatal onset of epilepsy [24]. Mitochondrial dysfunction has been extensively claimed to have a contributing factor also in other neurological disorders, for example, amyotrophic lateral sclerosis (ALS) and Alzheimer disease's (AD). In AD, alterations in enzymes involved in oxidative phosphorylation have been reported in hippocampal neurons. In PD, mutations in proteins related to mitochondria have been identified and mitochondrial DNA mutations have been found in neurons of the *substantia nigra.* In ALS, the activity of some mitochondrial respiratory-chain enzymes is generally affected and mitochondrial-resident cell death proteins result altered. It is recently emerging that also alteration of mitochondrial quality control may have a leading role in the neurodegenerative process. Indeed, altered mitochondrial biogenesis and/or mitochondrial clearance of damaged mitochondria through autophagy (mitophagy) have been detected in degenerating neurons.

A growing body of evidence suggests that restricting calorie intake has a strong beneficial effect on neuronal function. In particular, caloric restriction (CR) is now widely considered a dietary regimen that may delay brain senescence and prevents neurodegeneration. It has been suggested that many of the beneficial effects of CR not only derive from its ability to regulate NAD^+^ and AMP cellular levels but also to modulate ROS and NO production ultimately leading to efficient mitochondrial activity [25]. Interestingly, NO and ROS may regulate via a redox-dependent mechanism PGC-1*α* both at expression and activity level [14, 25–27].

Here, we review the current knowledge and recent data related to the role of PGC-1*α* and CR in neurodegeneration and illustrate the potential underlying signaling pathways characterizing the MN-C. We also discuss how such pathways might serve as targets for nutritional intervention to prevent ageing and age-related neurodegenerative diseases.

## 2. PGC-1*α* ****in the Orchestration of ****Mitochondrial-Nuclear Communication

A key factor in the MN-C is the transcriptional coactivator PGC-1*α*. Like all transcriptional coactivators, PGC-1*α* modulates the activity of several transcription factors without directly binding to DNA and without having any known intrinsic histone deacetylase or other enzymatic activities. The activity of PGC-1*α* only consists in mediating the interaction between transcription factors and RNA polymerases so influencing gene expression [28]. PGC-1*α* was originally identified as a transcriptional regulator of PPAR*γ* capable of stimulating UCP-1 expression in brown adipose tissue [29]. Subsequently, PGC-1*α* has been shown to coactivate a number of nuclear receptors, including PPARs, HNF4*α*, GR, and ERR*α*, in addition to transcription factors such as NRF-1, MEF2C, FOXO1, and YY1 [28, 30]. This wide variety of binding partners empowers PGC-1*α* to induce context-specific coordinated sets of gene expression that are tailored to the immediate physical demands of the organism, including energetic failure (nutrient limitations/physical exercise) and oxidative stress.

Recent studies indicate that regulation of cellular oxidative capacity through enhancing mitochondrial biogenesis may be beneficial for neuronal recovery and survival in human neurological disorders. Indeed, PGC-1*α* protein is very abundant in neural progenitor cells and newly generated neurons in the embryonic and early postnatal brain, suggesting a role for PGC-1*α* in the dynamic processes of neuroplasticity occurring during this time period [31]. In mitochondrial biogenetic pathways, PGC-1*α* is able to induce the expression and activation of NRF1 and 2 transcriptional factors implicated in the induction of mitochondrial genes including TFAM, which in turn coordinates the replication and the expression of genes located on mtDNA [32]. Consistent with its role as a master regulator of mitochondrial biogenesis, PGC1*α* expression closely correlates with mitochondrial content and is induced upon different stimuli eliciting a shift from glycolytic to oxidative metabolism. In particular, PGC-1*α* induces thermogenesis in brown adipose tissue, fiber-type switching in skeletal muscle, and fatty acid *β*-oxidation, along with gluconeogenesis, in the liver [33]. More recently, it was discovered that in the intestine PGC-1*α* governed the intestinal epithelium cell fate by promoting enterocytes differentiation and apoptosis by virtue of its capacity to enhance mitochondrial respiration and consequently ROS production [34]. Moreover, PGC-1*α* in neuronal cells is able to induce mitochondrial biogenesis and elevate basal respiration [35]. Several lines of evidence link PGC-1*α*  alteration and neurodegeneration and it has been reported that PGC-1*α* is capable of improving or rescuing mitochondrial dysfunctions implicated in the pathogenesis of several age-related diseases including neurodegenerative diseases [36–38]. Very recently, it was demonstrated that PGC-1*α* is downregulated in postmortem brains of PD patients. This event is due to decreased E3 ligase activity of mutated Parkin impairing the proteasome-mediated degradation of a major transcriptional repressor of PGC-1*α* gene promoter (i.e., parkin-interacting substrate, PARIS) [38].

In a clinical context, being oxidative stress and mitochondrial dysfunction typical hallmarks of neurodegeneration, PGC-1*α* could be a useful tool to counteract or to limit the development of neurological disorders. Coherently with this hypothesis, PGC-1*α* null mice are prone to the development of neurodegeneration under treatment with PD-related drugs and upregulation of PGC-1*α* in mouse model of neurodegeneration ameliorates disease's symptoms [37, 39, 40]. Similarly, PGC-1*α* deficiency leads to behavior abnormalities, which are associated with axonal degeneration in the brain, especially in the striatum, a key region controlling body movements. The molecular basis of this axonal degeneration is not completely understood, even though impaired energy homeostasis and ROS dysmetabolism due to defect in mitochondrial function are likely the most prominent causes. A pathogenetic hypothesis could be that in neurodegeneration there is an inability of PGC-1*α* in sensing mitochondrial-produced ROS causing a break in MN-C. It was demonstrated that the expression of this coactivator could be induced by ROS and this event is able to stimulate redox transduction pathways culminating in the expression of antioxidant genes (e.g., GPx1 and MnSOD) [37]. So, PGC-1*α*, promoting mitochondrial biogenesis, which unavoidably increases ROS level through the ETC, is also able to induce antioxidants expression, thus maintaining normal redox status in response to changes in oxidative capacity. For this reason, PGC-1*α*-way of action has been defined as “a clean energy program” [41]. Besides modulating PGC-1*α* expression, mitochondrial ROS are also able to modulate PGC-1*α* activity [37, 42]. In particular, it has been found that ROS-induced p38 mitogen-activated protein kinase (p38 MAPK) cascade culminates in the phosphorylation of PGC-1*α* at three residues (T262, S265, and T298) leading to PGC-1*α* increased stability and half-life. It is interesting that these phosphorylations occur in a region previously shown to play an important regulatory role in PGC-1*α* binding to transcription factors including many nuclear receptors and NRF-1. The docking of these transcription factors causes a conformational change that accelerates the binding of other transcriptional effector proteins into this complex, including CBP/p300 and SRC-1 resulting in a more efficient PGC-1*α*-mediated stress resistance [43]. Anderson and colleagues have shown a subcellular redistribution of PGC-1*α* upon oxidative stress. After the treatment of cells with an oxidative insult, GSK3*β* is activated and phosphorylates PGC-1*α* leading to its intranuclear digestion by the proteasome system. This regulatory pathway allows that the activation and degradation of PGC-1*α* can be independently controlled. Moreover, this study demonstrated that PGC-1*α* represents a good candidate for the regulation of cellular metabolic activity through the coordination of MN-C upon stress conditions [44]. In this scenario the comprehension of the molecular mechanisms that control cellular fate through the mitochondria-nucleus network could be useful to explore new strategies and pharmacological treatments for neurodegenerative diseases characterized by ROS imbalance.

Also other mitochondrial mediators can modulate PGC-1*α* activity thus affecting its ability to promote compensatory responses under stress conditions. Indeed, PGC-1*α* activity can be finely tuned in response to different metabolic situations and the metabolic sensors AMPK and SIRT1 have been described to directly affect PGC-1*α* activity through phosphorylation and deacetylation, respectively [45]. These sensors would act as integrative nodes in MN-C determining which transcriptional responses will be operative in order to adapt to the environmental conditions.

SIRT1 is one of the mammalian homologues of yeast, the Sir2 protein, and the founding member of the sirtuin gene family and mediates NAD^+^-dependent deacetylation of specific target substrates. As the cellular redox balance of NAD^+^ and NADH is highly related to mitochondrial activity, it has been postulated that SIRT1 could act as a sensor that directly connects metabolic perturbations with transcriptional outputs, as it was initially characterized as a histone deacetylase [46]. Although our understanding of mammalian SIRT1 biology is still surprisingly weak, there is compelling evidence implicating it as a major factor controlling metabolic homeostasis. First of all, during the last decade, a number of reports have shown that SIRT1 is not just a histone deacetylase. In fact, SIRT1 can directly interact and regulate the activity of coregulators, including PGC-1*α*. In particular, it has been shown that SIRT1-mediated PGC-1*α* deacetylation enhances its transcriptional activation, and mutation of the acetylation sites to arginine, which mimics the deacetylated state, markedly increases basal PGC-1*α* transcriptional activity [28]. In relation to this aspect, several evidences demonstrate that activation or overexpression of SIRT1 could be used to compensate neuronal mitochondrial loss [47]. On the contrary, energetic state linked to an increased NADH/NAD^+^ ratio are able to promote an hyperacetylation of PGC-1*α* mediated by general control nonderepressible 5 (GCN5) and steroid receptor coactivator 3 (SRC-3), thus diminishing PGC-1*α*  transcriptional activity. Moreover, when PGC-1*α* is deacetylated by SIRT1 and sequestered into the nucleus, an effect on transcriptional activity of PGC-1*α* mitochondrial target genes (nuclear genes encoded for component of ETC) is observed.

AMPK constitutes a molecular hub for cellular metabolic control, common to all eukaryotic cells. Numerous reports have established how AMPK responds to changes in the AMP/ATP ratio as a measure of cellular energy levels. AMPK activation is highly relevant for the transcriptional adaptation to physiological situations of energy demand. Mice expressing a dominant-negative form of AMPK cannot increase mitochondrial biogenesis in response to energy deprivation in skeletal muscle [48, 49]. On the other hand, in mice overexpressing an activated form of the AMPK, the expression of genes controlling mitochondrial activity is induced [50]. These results strongly confirm a potential role of AMPK in the MN-C. In fact, AMPK constitutes a major regulator of basal mitochondrial gene expression as well as mitochondrial gene expression upon energy stress. Interestingly, there is a strong overlap in the genes transcriptionally regulated by AMPK and those by PGC-1*α*, hence suggesting that PGC-1*α* might be an important mediator of AMPK-induced gene expression. Supporting this hypothesis, AMPK activation leads to increased PGC-1*α* expression and AMPK requires PGC-1*α* activity to modulate the expression of several key players in mitochondrial process [51, 52]. However, a closer link has been provided by findings showing that AMPK can directly interact and phosphorylate PGC-1*α*. It has been revealed that AMPK activation (phosphorylation on ser 172) seems to increase transcriptional activity of PGC-1*α*, even though the reasons why, where, and how that happens are still elusive. On the opposite mechanisms, diet rich in energy, which is associated with an increased ATP/AMP ratio, reduces AMPK phospho-activation and this event is associated with a decreased activation of PGC-1*α* [52]. In relation to the fact that neurons are highly metabolically active, and have poor capacity for nutrient storage, the role of AMPK in the development, function, and maintenance of the nervous system has only recently gained attention.

## 3. MN-C during Caloric Restriction: ****Implication in Neurodegenerative Diseases

Nutrient stress is generally considered from the standpoint of how cells detect and respond to an insufficient supply of nutrients to meet their bioenergetic needs. However, cells also experience stress as a result of nutrient excess, during which there is an increased ROS production combined with augmented ATP and NADH levels [53]. Therefore, it is possible that the resulting energetic overload can generate cell responses correlated with pathophysiological conditions. Sparks and coworkers [54] have shown that a high-fat diet (HFD) was linked to down-regulation of PGC-1*α* protein and mRNA, as well as expression of genes encoding proteins of complexes I, II, III, and IV of the ETC [55]. These changes were recapitulated and amplified in a murine model of HFD, along with decreases in PGC-1*α* and cytochrome *c* protein. A hypothetical model by which nutrient excess, and in particular a chronic nutrient excess, can induce mitochondrial dysfunction could be related to an engulfment in MN-C pathways. In particular, the increase in ATP and NADH induces a reduction in AMPK activation and SIRT1 deacetylating potential, thus negatively modulating PGC-1*α* activity. This status, in concomitance to a persistence of high ROS levels could induce a switch from cell resistance to cell damage, promoting a degenerative process especially in neurons. By far the greatest risk factor for neurodegenerative disease is ageing, and the age-dependence of such pathologies has led to experiments investigating the therapeutic effects of CR on neurodegeneration. CR is usually defined as a moderate reduction, generally 20–40%, in caloric intake compared with *ad libitum* feeding, without compromising the basic nutritional needs [56, 57]. Several studies have reported the inverse relationship between caloric intake and risk of neurodegeneration [57], fitting well within the context of the CR paradigm, one of the most robust in gerontology [58]. In Rhesus monkeys, CR lowered the incidence of aging-related death, diabetes, cancer, cardiovascular disease, and brain atrophy [59]. A significant increase in survival in CR monkeys as well as attenuation of the age-related declines in brain volume in selected regions was found. Thus, in specie closely related to humans, CR has shown promise as an intervention that could retard brain aging and neurodegeneration.

Although the mechanisms by which CR performs these effects are still not well understood, a possible role for MN-C could be envisaged [45, 60]. As aforementioned, brain strongly depends upon the functions of mitochondria, which can dialog with nucleus through some metabolic intermediaries (ATP and NAD^+^) and ROS. In the energetic context, neurons are highly specialized cells requiring large amounts of ATP and about 90% of this metabolite generated in the brain is synthesized in mitochondria *via* oxidative phosphorylation [61]. In neurons it has been demonstrated that CR-mediated AMPK activation brings a strong neuroprotective function by regulating mitochondrial biogenesis [62, 63]. In particular, a potential mechanism, by which CR is able to require AMPK-mediated mitochondrial biogenesis, could be linked to a metabolic shift from carbohydrate to fat utilization, which produces less ATP by the ETC [64]. Thus, it is possible that the observed mitochondrial biogenesis during CR compensates for the reduction in ATP production (increased AMP/ATP ratio)/reducing equivalent when fat is used as an energy source. In a molecular context, the mechanisms by which the activated AMPK regulates mitochondrial biogenesis are beginning to be elucidated. However, by using *in vivo* models it is emerging that nuclear PGC-1*α* could be a crucial mediator of AMPK action on mitochondrial-nuclear loop [65]. Interestingly, even in human subjects engaged in CR or CR plus exercise it has been demonstrated an AMPK-mediated increase in PGC-1*α* levels [66]. Similarly, a cell culture model of CR, in which cells are cultured with serum from calorie-restricted humans or rats, exhibited an increased PGC-1*α* expression with a parallel enhancement in mitochondrial efficiency [67]. However, the beneficial effects of CR on neuronal function may also rely on the intervention of other mediators of MN-C; that is, NAD^+^ [64]. In light of this, CR may be able to retain SIRT1 activity due to the recent evidence demonstrating that NAD^+^ levels in liver increase with fasting [68], and that changes in the NAD^+^/NAM-ratio *in vivo* critically influence cellular responses to CR in mammals by modulating SIRT1 activities [69]. The SIRT1 deacetylation reaction consumes NAD^+^ with consequent release of 2′-Oacetyl-(ADP) ribose NAM [70], which in turn inhibits SIRT1 by a negative loop. Thus, the recycling of NAM back to NAD^+^ by the enzyme nicotinamide phosphoribosyltransferase is absolutely crucial for the maintenance of cellular NAD^+^ and for maintaining SIRT1 functions [71]. An important major target for SIRT1 deacetylation activity is PGC-1*α*, which has been shown to be deacetylated in a model of neurodegeneration and in the brain in response to resveratrol treatment. This natural compound is believed to exert beneficial effects that nicely mimics those related to CR. The way of action of resveratrol on cell metabolism is a still-debated matter. The most fascinating theory is that resveratrol can increase the affinity of SIRT1 for its nuclear acetylated substrates or augment NAD^+^/NADH ratio [72]. The resulting PGC-1*α* activation by resveratrol represents a signaling pathway promoting the slowdown of the neurodegenerative process [73]. This neuroprotective action is very likely because PGC-1*α* induces mitochondrial activity, being neurodegeneration linked to mitochondrial failures. However, other SIRT1-independent beneficial action of resveratrol could be hypothesized. Indeed, resveratrol is able to selectively inhibit the activity of MEK1/2, responsible for the activation of the down-stream ERK1/2 [8]. Autophagic cell death seems to be a contributing factor in the neuronal loss observed in neurodegeneration and this event is mediated by ERK1/2-governed signaling pathways [74, 75]. Therefore, besides the SIRT1-mediated activation of PGC-1*α*, it is plausible to postulate that the beneficial effect of resveratrol on neuronal homeostasis could be partially due to the inhibition of ERK1/2-mediated autophagic cell death.

In the study of Anderson et al. [44], PGC-1*α* translocates to the nucleus during CR and here it is activated by SIRT1 and subsequently phosphorylated by GSK3*β*, priming it for ubiquitination and nuclear degradation. The net result is a transient PGC-1*α* activation enabling a rapid transcriptional stress response that is quickly shuttled off by degradation. However, under chronic stress conditions such as prolonged CR, GSK3*β* does not phosphorylate PGC-1*α*, so that PGC-1*α* is perpetually active in the nucleus, facilitating a sustained increase in transcription of genes involved in mitochondrial function [44].

Recently, Fusco et al. [76] have reported that the effects of CR on neuronal plasticity, memory, and social behavior are abolished in mice lacking CREB, a molecular mediator which can be implicated in MN-C. It was shown that CREB deficiency drastically reduces the expression of SIRT1 and the induction of genes relevant to neuronal metabolism and survival in the cortex and hippocampus of dietary-restricted animals. Biochemical studies reveal a complex interplay between CREB and SIRT1: CREB directly regulates the transcription of SIRT1 in neuronal cells by binding to SIRT1 chromatin; SIRT1, in turn, is recruited by CREB to DNA and promotes CREB-dependent expression of the target gene PGC-1*α*. Accordingly, expression of these CREB targets is markedly reduced in the brain of SIRT1 KO mice that are, like CREB-deficient mice, poorly responsive to CR [76]. The beneficial effect of CR on lifespan is likely linked to the increase of NO production. Specifically, it was demonstrated that CR increases endothelial NOS (eNOS) and neuronal NOS (nNOS) [25, 76]. The raise of NO levels is the mediator of mitochondrial biogenesis that is paralleled by the increased oxygen consumption, ATP production, and an enhanced expression of SIRT1. Only few reports indicate that CR may induce increased GSH levels or dampen the effects of ageing on GSH decrease [77, 78]. Controversially, we demonstrated that GSH is an efficient physiological buffer of NO reducing its bioavailability [13]. Unpublished data from our laboratory indicate that nutrient limitation by fasting is instead associated with decreased level of GSH in brain, which may potentiate the beneficial action of NO. With this in mind, the effective increased level of brain GSH during CR certainly merits further investigation. 

**Figure 1:**
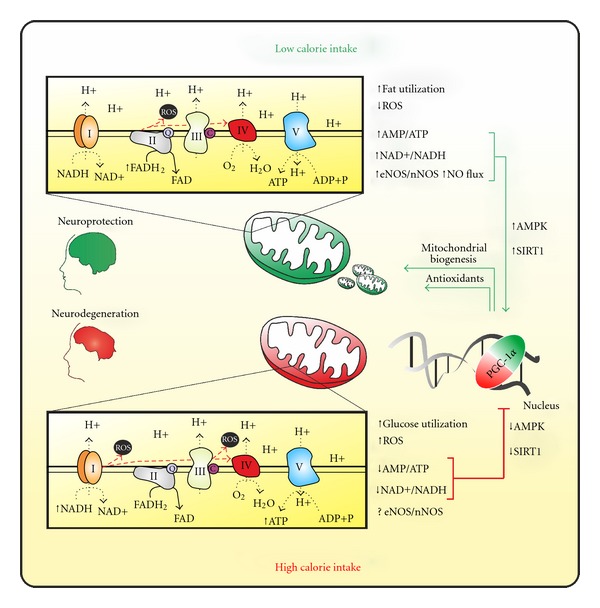
Schematic model of the effect of calorie intake on PGC-1*α*-dependent mitochondrial activity and its implication in neuronal health.

ROS can be generated by a variety of enzymes of oxidative metabolism among which the ETC is the most significant source. But during CR, how might the biogenesis of mitochondria correlate with a reduction of ROS? An important factor reducing ROS is the increase of antioxidant levels that often associates with CR and that is mediated by PGC-1*α* as discussed above [41]. Another potential mechanism could be that, when fat is used as energy source, the frequency with which electrons enter the ETC bypassing complex I is increased. Given that complex I is one of the primary source of ROS, this could justify ROS reduction during CR. A schematic model of how the rate of caloric intake can modulate mitochondrial biogenesis and affect neuronal homeostasis is reported in Figure 1.

## 4. Recent Advances in PGC-1*α* ****Biology: ****The Extranuclear Forms

For long time PGC-1*α* has been prevalently considered a nuclear-resident protein prevalently acting on nuclear gene expression. It has been recently described that PGC-1*α*  can have an extra-nuclear localization. Chang and colleagues have identified a novel, biologically active 270 amino acid isoforms of PGC-1*α* (NT-PGC-1*α*) produced by alternative splicing. In contrast to full-length PGC-1*α* (~90 kDa), which is relatively short-lived (~2.3 h) due to rapid targeting to ubiquitin/proteasome-mediated proteolysis in the nucleus, NT-PGC-1*α*  (~38 kDa) is more stable and predominantly sequestered in the cytoplasm. Upon increase of cAMP levels, that is, during cold exposure of brown adipose cells, NT-PGC-1*α* efficiently accumulates within nuclei and its recruitment to the promoters of PGC-1*α* target genes is strongly enhanced. Moreover, the phosphorylation of NT-PGC-1*α* by PKA inhibits the nuclear export and a greater proportion of NT-PGC-1*α* remains into nucleus [79], where it mediates the transcription of many genes that are both distinct from and common to those that are regulated by PGC-1*α*. These data suggest that the expression of NT-PGC-1*α* instead of full-length PGC-1*α* would be important for a more efficient and complete induction of a set of genes that are transcriptionally regulated by PGC-1*α* isoforms [79, 80], thus assuring a prolonged induction of PGC-1*α*-regulated genes. Unfortunately the presence and the role of NT-PGC-1*α* in other cell types including neurons are unascertained yet. Given that PGC-1*α* deregulation has been found as the cause of neuronal death, the role of NT-PGC-1*α* in neurodegenerative diseases needs to be clarified.

**Figure 2:**
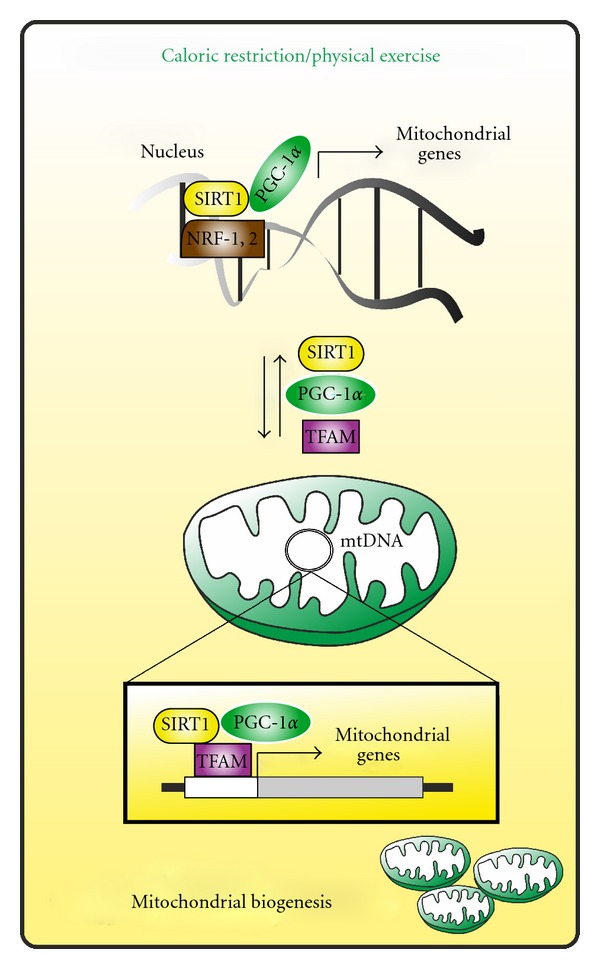
Hypothetical role of extra-nuclear forms of PGC-1*α*  and SIRT1 in mitochondrial biogenesis. Upon energetic stress conditions (e.g., physical exercise, caloric restriction) PGC-1*α*  and SIRT1 are implicated in the expression of mitochondrial genes at the nuclear level. Concomitantly, PGC-1*α*  and SIRT1 might migrate into mitochondrial matrix wherein, by interacting with TFAM, mediate the transcription of mtDNA-encoded mitochondrial genes, thus inducing mitochondrial biogenesis.

The extra-nuclear localization of full length of PGC-1*α* was also demonstrated in our laboratory [81]. Specifically, we showed that PGC-1*α* localizes together with SIRT1 within mitochondria, wherein they indirectly associate with the regulatory D-loop region of mtDNA. Such region is the site of initiation of mtDNA replication and transcription and here they interact with TFAM (which specifically binds to D-loop). On the basis of these findings we suggested that SIRT1 and PGC-1*α* likely participate in the transcription/replication of mtDNA. We postulated that SIRT1 could activate TFAM and PGC-1*α* by deacetylation, and PGC-1*α* in turn coactivates TFAM. With this in mind the group of Safdar successively showed that upon acute energy demands; that is, endurance physical exercise, PGC-1*α* is increased and localizes both into nuclear and mitochondrial fractions of skeletal muscle cells [82]. With regard to mitochondrial PGC-1*α*, they demonstrated that an increased interaction of PGC-1*α* with TFAM was operative, resulting in enhanced TFAM coactivation and more efficient mtDNA transcription. This mitochondrial event is accompanied by the augmented activity of the nuclear PGC-1*α* allowing the concomitant transcription of nuclear-encoded mitochondrial genes. Therefore, altogether these recent findings put PGC-1*α* at the center stage of MN-C being able to contemporarily promote the transcription of nuclear and mitochondrial-encoded genes upon specific metabolic needs such as during physical exercise and CR (Figure 2). Notwithstanding, the mitochondrial import of PGC-1*α*, as well as of SIRT1, remains an unsolved and intricate question. Indeed, PGC-1*α* and SIRT1 do not contain the canonical mitochondrial targeting signal needed for the mitochondrial import [83]. However, data from proteomic analyses indicate that nearly half of the nuclear-encoded proteins residing within mitochondria may be imported by exploiting cryptic internal signals [84] and this could be the case of PGC-1*α* and SIRT1. The precise stimuli dictating the mitochondrial redistribution of PGC-1*α* and SIRT1 is still unknown and will be of great interest for future studies. Given that chronic physical exercise is associated with oxidative stress and a substantial decrease and oxidation of GSH [85], a role of ROS and/or NO could be postulated as determinants of such redistribution. For instance these second messengers could regulate nuclear and mitochondrial shuttling of PGC-1*α* by posttranslational modifications comparable to those necessary for its full activation. Similar pathways managing the nuclear-mitochondrial shuttling of PGC-1*α* could be operative during CR. Actually, PGC-1*α* activators that is, AMPK, p38MAPK, and SIRT1 can be modulated by NO/ROS signaling as well as by CR [86–89].

## 5. Conclusion and Perspectives

Mitochondria and nuclei are interconnected through a communicative loop. Their cross-talk operates broadly at two levels: one mechanism involves cellular responses to changes in the functional state of the mitochondria itself, where mitochondrial mediators are able to modulate a set of nuclear transcription factors, or coactivators, impinging the expression of both nuclear and mitochondrial proteins fundamental in maintaining cell metabolism. In the opposite route, several nuclear-encoded proteins can “talk” with mitochondria inducing adaptive responses. Mitochondrial-nuclear reciprocal signaling is a central question that needs to be answered to understand the molecular mechanisms, assuring mitochondrial activities and functions. Considering the negative impact of mitochondrial dysfunction on cell function and human health, the comprehension of the molecular mechanisms underlying MN-C continues to be a fundamental issue and matter of research.

The dysfunction of mitochondrial metabolism is the main contributing factor in the etiology/progression of many neurodegenerative diseases. It is emerging that altered bioenergetics is triggered by a deregulation of mitochondrial genes expression. Impairment of SIRT1 and/or PGC-1*α*  activity takes center place in such deregulation being fundamental regulators of mitochondrial metabolism and ROS homeostasis at transcriptional level. The possible direct contribution of altered SIRT1 and/or PGC-1*α*  activity in neurodegenerative diseases has been demonstrated by many *in vivo* and *in vitro* experimental models. Pharmacological or genetic manipulation of SIRT1 and PGC-1*α*  prevents neurodegeneration in animal and cellular models of ALS, AD, and PD [90, 91]. Interestingly, PGC-1*α*  has been directly linked to AD and PD pathogenesis, since it is significantly decreased in postmortem brains [38, 92].

The discovery of the involvement of SIRT1 and/or PGC-1*α*  in neurodegenerative diseases supports the use of therapies targeting the cellular metabolism and mitochondrial biogenetic pathway at SIRT1/PGC-1*α*  level. CR could be a promising nutritional strategy ultimately leading to PGC-1*α*-mediated mitochondrial biogenesis [73] and life-span extension. Consistent data corroborates the existence of cytoplasmic/mitochondrial forms of PGC-1*α*  and SIRT1, in which deregulation could be causative of neurodegenerative diseases characterized by mitochondria dysfunction. However, at least in recent studies, this issue remains underexplored. It is plausible to hypothesize that mitochondrial impairment in neurodegenerative disorders could be due not only to a defective levels/activities of PGC-1*α*  and SIRT1 at the nuclear but also at the mitochondrial level, with a significant negative impact on mtDNA transactions (replication and expression), mitochondrial number, and metabolic function. This can be due to the downregulated basal level of these proteins or to a deficit in the mitochondrial import mechanisms. In this regard, similarly to physical exercise, energetic failure caused by CR may have a functional role not only in promoting PGC-1*α*  and SIRT1 activity at the nuclear genome but also in enhancing mtDNA transcription, thus, likely resulting in a more efficient mitochondrial biogenesis and increased lifespan.
